# Evaluation of machine learning classifiers for predicting essential genes in *Mycobacterium tuberculosis* strains

**DOI:** 10.6026/973206300181126

**Published:** 2022-12-31

**Authors:** Monish Mukul Das, Keka Sarkar

**Affiliations:** 1 Department of Computer Science and Engineering, University of Kalyani, Kalyani, Nadia - 741235; 2 Department of Microbiology, University of Kalyani, Kalyani, Nadia - 741235

**Keywords:** Essential gene, *Mycobacterium tuberculosis*, Genome extraction program, Deep Neural Networks (DNN), Support Vector Machine (SVM), Decision Tree(DT), Genetic Algorithm, Area under the Receiver operating characteristics curve (AUC)

## Abstract

Accurate investigation and prediction of essential genes from bacterial genome is very important as it might be explored in effective targets for antimicrobial drugs and understanding biological mechanism of a cell. A subset of key features data obtained from 14 genome sequence-based features of 20 strains of *Mycobacterium tuberculosis* bacteria whose essential gene information was downloaded from ePath and NCBI database for mapping and matching essential genes by using a genome extraction program. The selection of key features was performed by using Genetic Algorithm. For each of three classifiers, 80%, 10% and 10% of subset key features were used for training, validation and testing, respectively. Experimental results (10-f-cv) illustrated that DNN (proposed), DT, and SVM achieved AUC of 0.98, 0.88 and 0.82, respectively. DNN (proposed) outperformed DT and SVM. The higher prediction accuracy of classifiers was observed because of using only key features which also justified better generalizability of classifiers and efficiency of key features related to gene essentiality. Besides, DNN (proposed) also showed best prediction performance while compared with other predictors used in previous studies. The genome extraction program was developed for mapping and matching of essential genes between ePath and NCBI database.

## Background:

The genomic information obtained from different *Mycobacterium tuberculosis* strains has shown higher genetic diversity corresponding to patterns of human migration, which suggests the co-evolution of distinct lineage with various human populations [1]. However, in addition to three virulence factors [2] the mycobacterial cell wall[3] is also very important in the pathology of M. tuberculosis. The active drug efflux systems, superfamilies of enzymes, and genes are involved in the drug resistance activity of M. tuberculosis [4]. Furthermore, the genes required to be critical for survival, development, and proliferation are considered to be essential and these are effective targets for antimicrobial drugs [5]. Essential genes are very much significant in understanding actual source of life and evolutionary relationships among different organisms and are believed to be evolved more slowly than non-essential genes [6]. However, essential gene identification in pathogens using genetic features across genome, requires sophisticated experimental strategies which are often time-consuming, laborious, costly, and have some limitations [7]. A number of machine learning methods, have been suggested in predicting gene essentiality by using various genomic features and strategies [8]. The applicability of computational methods which require features of gene ontology annotations, gene-expression, functional domain and network topology, rely on obtainability of experimental data [9]. Thus, many scientists worked on genome sequence features in predicting gene essentiality [7][10] [11][12]. Very recently, Xu et al. [13] used key features derived from sequence for prediction of essential genes in prokaryotes by using artificial neural networks. In 2018, Azhagesan et al [14] used SVM for classification of essential genes across the diverse bacterial species by using network-based features and the classifier achieved better AUC score (0.847). Liu et al. [10] made an expensive study on 31 diverse bacterial species based on SVM by using sequence based features but their prediction result was not satisfactory due to biases in feature and redundancy. Again, Song et al. [15] used another effective essential gene predictor, ZUPLS which was evaluated on sequence information based features in addition to other kinds of features and they achieved better prediction performance of predictor. In 2011, Deng et al. [16] used 13 important features out of 28 sequence and other categories of features for their predictor consisting of four machine learning algorithms which yielded best performance in predicting gene essentiality of four organisms. Afterwards, Cheng et al. [17] reported better performance of their computational method comprising of three machine learning algorithms by using 16 features obtained from sequence information, gene-expression and network topology characters of 21 organisms. Nigatu et al. [9] used a machine learning model which includes Random Forest in predicting gene essentiality and they achieved very good results with high AUC scores by using 81 information theoretic features derived from DNA sequences of 15 organisms. As many features may affect generalizability and accuracy of predictors, the features selection is of great importance in machine learning methods for classification function [10]. The genetic algorithm in combination with machine learning algorithms acts as a relevant and unique feature selection technique which has been frequently used in Cancer prognosis [18] and other biological fields [17]. In this paper, genetic algorithm(GA) based on random forest(RF) classifier was explored to screen only relevant and unique key features from original sequence features. To analyse accuracy in predicting essential genes of M. tuberculosis drug resistant strains, 10-fold cross validation was performed with three machine learning algorithms - DT,SVM and DNN(proposed) by using key features subset.

## Methodology:

### Retrieval of Genome sequence data:

The information about essential genes (Gene locus IDs) of 20 strains of M. tuberculosis bacteria was taken from e-Path hypothetical essential gene database [19]. The genome sequences were downloaded simultaneously from NCBI Gene Bank. The data from e-Path and NCBI were then mapped by using gene identification number. Genes of NCBI which matched with genes tagged as essential in e-Path, were marked as essential and the rest mismatched gene sequences were labelled as non-essential. A genome extraction program was developed and executed for mapping and matching essential genes between e-Path and NCBI Gene Bank (Figure 1 - see PDF).

The results (Table 1 - see PDF) illustrates the details of essential and non-essential genes of M. tuberculosis strains and it was seen that essential and non-essential gene ratio stands 1:2.9 which indicated an imbalanced dataset. Thus with a view to enhance prediction performance of three classifiers, strategies for down-sampling and redundancy reduction in non-essential genes(majority class) were applied as imbalance datasets creates problems for classifiers [11]. Accordingly, redundancy reduction by homology clustering in majority class was made using CD-HIT program [20]. After that random under sampling of majority class data was performed and an amount of randomly selected non-essential genes equal to all essential genes was used for generation of sequence based feature dataset. The unbiased randomness of final dataset was ensured by repeating the process of random sampling 10 times [8].

### Generation of sequence feature dataset:

In respect of each gene in the balanced dataset, 14 sequence based features including amino acid length and codon frequencies were extracted using CodonW [21], a multivariate analysis program which calculates indices of Codon and Amino acid usage. The sequence based 14 features were extracted and these include, codon adaptation index (CAI), frequency of optimal codons (Fop), codon bias index (CBI), effective number of codons(Nc), GC content of gene(GC), G+C content 3rd position of synonymous codons (GC3s), base composition at silent sites (G3s, C3s, A3s T3s), length of system amino acids(L_sym), length of amino acids (L_aa), hydropathicity of protein(Gravy), frequency of aromatic amino acid(Aromo) which are extensively used for gene essentiality. Feature data preparation and computational methods were performed using Python 3.5.2.

### Selection of Critical features for sub dataset:

The generalization ability and accuracy performance of prediction models are straight way related to selection of key features [10][13][16] [17]. In this study, key features were selected from most common sequence based features dataset by using Genetic Algorithm based on Random Forest Classifier and the result includes 10 selected features namely CAI, CBI, GC, G3s, C3s, A3s, T3s, L_aa, Gravy and Aromo. Genetic algorithm in combination with random forest classifier was executed through scikit-learn-genetic-opt module of python to screen only key features from original features derived from sequence [17].

### Classifiers used in current work:

Considering the prediction of essential genes as a binary classification, three Machine Learning classifiers namely Lib SVM - RBF, C 4.5 DT and a newly designed DNN with MLP were evaluated on subset of key features and the findings showed no logical contradiction with previous works [9][11] [16][17]. DT was used in data mining for classification and regression as a tree. It was incorporated in the present study as it permits induction of a set of classification rules. It generates a framework of measure the values of outcomes. In this classification method SVM was executed as it may work even while there is some biasness in training datasets. DNN was used as it works better for mapping non-linearity of data even while non-linearity is on higher side. Besides, it has got self-adaptability and it does not require to be reprogrammed. Machine learning classifiers were implemented using Scikit-learn 1.1.2, a python library. In addition, MLP networks have been investigated with great success in many biological problems [22]. In the present study, rectified linear unit (ReLU) was also executed as activation function in DNN and softmax activation function was used to predict probability of samples whether it belongs to essential or non-essential gene class.

Relu = f(x) = max(0,x) (1)

Softmax(z)_I_ = Exp(Z_i_)/Σ_i_=1to K Exp(Z_i_) (2)

Here, Z_i_ represents the i^th^ element of input to the softmax function.

### Evaluation of classifiers:

The classifiers were trained with 80% of total subset key features and 10% data was used for validation. The testing of classifiers was executed with remaining 10% of subset key features. The entire training sub dataset were divided into 10 equal divisions and 10-f-cv was performed with three classifiers by using subset key features. The accuracy metrics were calculated from the confusion matrix computed for each of ten divisions of dataset during training and the mean of the accuracy metrics was calculated to determine final accuracy metrics after 10-fold cross validation (10-f-cv). Area under Receiver operating characteristics(AU-ROC) curves were generated with three classifiers using subset key features and results showed the numerical score of AUC, S_n_(Sensitivity), S_p_(Specificity), PPV, Accuracy(Acc), NPV though AUC score is considered as primary evaluation measure for classifiers performance. The other performance measures are appended below:

*Sensitivity = True Positives/True Positives + False Negatives (3)*

*Specificity = True Negatives/ False Positives + True Negatives (4)*

*Positive Predictive Value (PPV/Precision) = True Positives/True Positives + False Positives Negative Predictive Value(NPV)=True Negatives/True Negatives + False Negatives (6)*

*Accuracy = TruePositives+TrueNegatives/True Positives+False Negatives+True*

*Negatives+False Positives (7)*

## Results and Discussion:

In this study, three classification algorithms - DT, SVM and DNN were explored with a view to evaluate the performance of each classifier with key features and analysed the impact of key features selection on the accuracy of classifiers. It was observed from Table 2(see PDF) that DNN was highly sensitive to key feature selection, where SVM and DT showed less sensitivity in accuracy with key features. However, DNN outperformed SVM and DT in respect of AUC. The average AUC scores of three predictors (Table 2-see PDF) did not contain any logical contradiction to previous work on prediction of essential genes of other bacteria [9][11][14] [16]. The predictive indexes of methods used in five selected representative papers were compared with that of three classifiers used in present study and the result illustrated that DNN achieved the best AUC score (0.98) among all predictors used in previous and present studies. Furthermore, the other additional measures like sensitivity (0.972) and PPV(0.960)scores of DNN in the present study were also much better than predictors of Liu et al. 2017. (0.715 and 0.243) based on 40 selected features, Azhagesan et al. 2018. (0.754 and 0.321) based on 267 selected features, Xu et al. 2020. (0.67 and 0.23) based on 29 key features and Hasan et al. 2020. (0.81 and 0.749) based on 89 selected features. It would not be out of the text to mention that 10-f-CV yielded very high AUC scores (0.86-0.93) and (0.69-0.89) in intra-organism and cross-organism prediction, respectively during the works of Deng et al. 2011 [16]. In addition, Nigatu et al. [9] also reported AUC scores of 0.92 and AUC score from 0.73 to 0.93 in cross-organism and intra-organism prediction, respectively. The result also illustrated that precision-recall for DNN was higher than that of SVM and DT and as such DNN is more capable of utilizing learning signals than SVM and DT. The classifiers evaluated on key features showed better result which suggested that genetic algorithm screened key features effectively. However, the application of DNN (proposed) on other bacteria could also be investigated in future work.

## Conclusion:

In this study, SVM, DT and a newly designed DNN with MLP approach were explored and evaluated on genome sequence based key features which were screened by using genetic algorithm based on Random Forest classifier. The DNN model (proposed) with highest prediction accuracy outperformed SVM and DT. Therefore, DNN model can be a valuable classifier for prediction of essential genes as potential drug targets. The results of the study justified the better generalizability of classifiers and effect of selected features on predictors' accuracy. There is an ample scope for further research work on the improvement of generalization ability of classifier by fine-tuning the discriminatory features.
